# Selective measurement of NAPE-PLD activity via a PLA_1/2_-resistant fluorogenic *N*-acyl-phosphatidylethanolamine analog

**DOI:** 10.1016/j.jlr.2021.100156

**Published:** 2021-11-26

**Authors:** Jonah E. Zarrow, Jianhua Tian, Brendan Dutter, Kwangho Kim, Amanda C. Doran, Gary A. Sulikowski, Sean S. Davies

**Affiliations:** 1 Chemical and Physical Biology Program, Vanderbilt University, Nashville, TN, USA; 2 Department of Pharmacology, Vanderbilt University, Nashville, TN, USA; 3 Vanderbilt Institute of Chemical Biology, Vanderbilt University, Nashville, TN, USA; 4 Department of Chemistry, Vanderbilt University, Nashville, TN, USA; 5 Division of Cardiovascular Medicine, Department of Medicine, Vanderbilt University Medical Center, Nashville, TN, USA

**Keywords:** NAPE-PLD, phospholipase A1, fluorescence, *N*-acylethanolamide, *N*-acylphosphatidylethanolamide, biothionol, tetrahydrolipstatin, diabetes, atherosclerosis, nociception, ABHD4, alpha/beta-hydrolase-4, AEA, *N*-arachidonyl-ethanolamide, Bith, bithionol, BMDM, bone marrow-derived macrophage, GP-NAE, glycerophospho-NAE, HEK293, human embryonic kidney 293 cell line, NAE, *N*-acyl-ethanolamide, NAPE, *N*-acyl-phosphatidylethanolamine, OEA, *N*-oleoyl-ethanolamide, PEA, *N*-palmitoyl-ethanolamide, PLC, phospholipase C, PLD, phospholipase D, THL, tetrahydrolipstatin

## Abstract

*N*-acyl-phosphatidylethanolamine (NAPE)-hydrolyzing phospholipase D (NAPE-PLD) is a zinc metallohydrolase enzyme that converts NAPEs to bioactive *N*-acyl-ethanolamides. Altered NAPE-PLD activity may contribute to pathogenesis of obesity, diabetes, atherosclerosis, and neurological diseases. Selective measurement of NAPE-PLD activity is challenging, however, because of alternative phospholipase pathways for NAPE hydrolysis. Previous methods to measure NAPE-PLD activity involved addition of exogenous NAPE followed by TLC or LC/MS/MS, which are time and resource intensive. Recently, NAPE-PLD activity in cells has been assayed using the fluorogenic NAPE analogs PED-A1 and PED6, but these substrates also detect the activity of serine hydrolase-type lipases PLA_1_ and PLA_2_. To create a fluorescence assay that selectively measured cellular NAPE-PLD activity, we synthesized an analog of PED-A1 (flame-NAPE) where the *sn*-1 ester bond was replaced with an *N*-methyl amide to create resistance to PLA_1_ hydrolysis. Recombinant NAPE-PLD produced fluorescence when incubated with either PED-A1 or flame-NAPE, whereas PLA_1_ only produced fluorescence when incubated with PED-A1. Furthermore, fluorescence in HepG2 cells using PED-A1 could be partially blocked by either biothionol (a selective NAPE-PLD inhibitor) or tetrahydrolipstatin (an inhibitor of a broad spectrum of serine hydrolase-type lipases). In contrast, fluorescence assayed in HepG2 cells using flame-NAPE could only be blocked by biothionol. In multiple cell types, the phospholipase activity detected using flame-NAPE was significantly more sensitive to biothionol inhibition than that detected using PED-A1. Thus, using flame-NAPE to measure phospholipase activity provides a rapid and selective method to measure NAPE-PLD activity in cells and tissues.

*N*-acyl-phosphatidylethanolamine (NAPE) hydrolyzing phospholipase D (PLD) is a zinc metallohydrolase that catalyzes the final step of *N*-acyl-ethanolamide (NAE) biosynthesis (1, 2, 3). NAEs, including *N*-arachidonyl-ethanolamide (AEA), *N*-oleoyl-ethanolamide (OEA), and *N*-palmitoyl-ethanolamide (PEA), play critical roles in the regulation of food intake, inflammation, and nociception (4, 5, 6, 7, 8, 9, 10, 11). Therefore, changes in NAPE-PLD activity may play an important role in the dysregulation of these processes that occur during cardiometabolic and neurological diseases. A facile assay to measure NAPE-PLD activity in cultured cells and tissues is needed to assess how changes in this activity may contribute to pathophysiology and to test the effectiveness of potential interventions targeting NAPE-PLD activity.

Current assay methods to measure NAPE-PLD activity are time consuming and/or lack selectivity for NAPE-PLD. NAPE-PLD activity has been most commonly measured by incubating radiolabeled NAPE with cells or cell homogenates, followed by extraction and separation of resulting NAE from NAPE by TLC (12). Depending on the cell type or tissue, this assay may not be selective for NAPE-PLD activity because it is usually carried out using radiolabeled NAPE with two *O*-acyl chains (diacyl-NAPE). Diacyl-NAPE can be hydrolyzed by alternative enzymatic pathways (6, 13, 14). For instance, alpha/beta-hydrolase-4 (ABHD4) exerts both PLA_1_- and PLA_2_-type phospholipase activity to hydrolyze diacyl-NAPEs to glycerophospho-NAEs (GP-NAEs) (6, 13, 15). These GP-NAEs can then be hydrolyzed to NAEs by the actions of glycerophosphodiesterases such as GDE1 and GDE4 (6, 15, 16). An assay that is more selective for NAPE-PLD activity can be achieved by using radiolabeled NAPE where the *sn*-1 and *sn*-2 ester bonds are replaced with ether bonds (diether-NAPE) to make it resistant to PLA_1/2_-type phospholipases including ABHD4. The results observed with diether-NAPE differ from those with diacyl-NAPE (17, 18, 19). Regardless of which radiolabeled substrate is used, the use of radioisotopes requires specialized licensing, handling, and waste disposal, and TLC is labor intensive and time consuming. Another widely used approach to measure NAPE-PLD activity is to add nonradiolabeled *N*-C17:0-phosphatidylethanolamine (C17:0 NAPE) and then measure the resulting change in the ratio of this NAPE and its respective C17:0NAE product by LC/MS/MS (20, 21, 22). This LC/MS/MS assay is at least as labor intensive, time consuming, and expensive as the radioisotope method, and unless diether-C17:0 NAPE is used as substrate, also suffers from lack of selectivity for NAPE-PLD. Therefore, either of these two approaches are too cumbersome to measure NAPE-PLD activity in the large number of samples that are often needed for time-course studies or for screening of the effects of various stimuli or small molecules in cultured cells and tissues.

A more recent alternative approach to measure NAPE-PLD activity utilizes PED-A1 or PED6, two commercially available fluorogenic compounds designed to measure PLA_1_ and PLA_2_ activity, respectively (Fig. 1) (22, 23, 24, 25, 26, 27, 28, 29, 30, 31). These two NAPE analogs are designed so that their hydrolysis by the intended phospholipase releases the dinitrophenol quencher moiety to allow detection of the BODIPY fluorophore. While both PED-A1 and PED6 allow for rapid and inexpensive measurement of NAPE-PLD activity in vitro, their sensitivity to PLA_1_ or PLA_2_ hydrolysis limits their effectiveness for selectively measuring NAPE-PLD activity in cultured cells or tissues. We therefore designed and synthesized a PED-A1 analog (flame-NAPE) where the *sn*-1 ester bond was substituted with an *N*-methyl amide bond anticipated to be resistant to phospholipase hydrolysis (Fig. 1) and showed that this analog can be used to rapidly measure cellular NAPE-PLD activity without interference from cellular PLA_1_ activity.

**Fig. 1:**
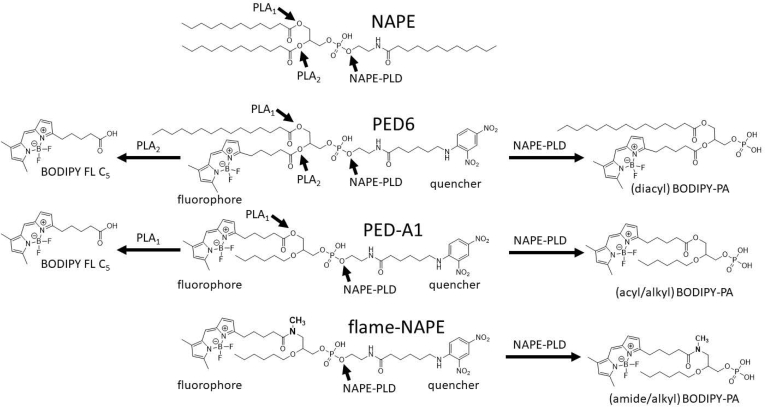
Flame-NAPE is a fluorogenic NAPE analog designed to be resistant to PLA_1/2_ hydrolysis but sensitive to NAPE-PLD hydrolysis. Endogenous NAPE can be hydrolyzed by various phospholipases, including PLA_1_, PLA_2_, and NAPE-PLD. PED6 is a fluorogenic NAPE analog where the dinitrophenol moiety of the *N*-acyl chain quenches the fluorescence of the BODIPY moiety on the *sn*-2 acyl chain. PLA_2_ hydrolysis of PED6 releases the quencher moiety to generate a fluorescent fatty acid (BODIPY FL C_5_), whereas NAPE-PLD hydrolysis generates a fluorescent phosphatidic acid (diacyl BODIPY-PA). PLA_1_ hydrolysis of PED6 releases the *sn*-1 fatty acid chain, but this does not generate fluorescence. PED-A1 is a fluorogenic NAPE where PLA_1_ hydrolysis generates BODIPY-FL C5, and NAPE-PLD hydrolysis generates a fluorescent phosphatidic acid (acyl/alkyl BODIPY-PA). The ether bond of the PED-A1 *sn*-2 chain is resistant to PLA_2_ hydrolysis. Flame-NAPE was designed to enable selective detection of NAPE-PLD by substituting an *N*-methyl amide bond for the *sn*-1 ester bond of PED-A1, thereby making it resistant to both PLA_1_ and PLA_2_ hydrolysis while retaining sensitivity to NAPE-PLD hydrolysis.

## Materials and methods

### Synthesis and purification of flame-NAPE

Synthesis and product verification for flame-NAPE were performed by the Molecular Design and Synthesis Center at Vanderbilt University as described in the supplemental data section. Briefly, commercially available (*R*)-glycerol acetonide was selectively functionalized and protected to provide the central amino diol. This enabled the sequential introduction of the *sn*-2 chain, phosphate, ethanolamine, *N*-acyl chain, and *sn*-1 chain. The final product was isolated by flash chromatography to >95% purity as indicated by LC/MS and NMR analysis.

### Acquisition of PED-A1 and flame-NAPE absorbance spectra

PED-A1 and flame-NAPE were prepared from dried stocks by dissolving in buffer containing 50 mM Tris-HCl, pH 8.0, ethanol, and DMSO (60:32:8 v/v/v), and the absorbance spectrum from 300 to 700 nm was recorded using an Eppendorf Biospectrometer kinetic UV-visible spectrometer.

### In vitro assays for characterization of PED-A1 and flame-NAPE hydrolysis by NAPE-PLD and PLA_1_

Recombinant mouse Nape-pld was expressed in *Escherichia coli* and purified as previously described (27). Purified *Aspergillus oryzae* Pla_1_ was purchased commercially (Sigma; L3295). PED-A1 and flame-NAPE were prepared in buffer containing 50 mM Tris-HCl, pH 8.0, ethanol, and DMSO (60:32:8 v/v/v).

#### *K*_*M*_ determination

55 μl of 50 mM Tris-HCl buffer (pH = 8) and 5 μl of 7% (w/v) *N*-octyl-β-d-glucoside solution (Millipore; 494459) was added to clear-bottom, black-walled, nontreated, 96-well plates (Thermo; 265301), followed by 5 μl of 73 μg/ml Nape-pld (final concentration of 4.56 μg/ml) or 5 μl of Tris-HCl buffer (for negative controls), and then 5 μl of test compound vehicle (1.6% DMSO [v/v] in Tris-HCl buffer) and incubated for 1 h at 37°C. To initiate the activity assay, 10 μl of ice-cold PED-A1 or flame-NAPE was added to every well with final concentration from 0 to 20 μM (six replicates per condition). Fluorescence (excitation/emission = 488/530 nM) was measured in a BioTek Synergy H1 plate reader at 37°C. Reaction rate was measured as Δfluorescence from *t* = 1 to *t* = 4 min, n = 6, mean ± SEM. *K*_*M*_ values were calculated in GraphPad Prism 7.0 (Graphpad Software L.L.C.) using nonlinear regression (allosteric sigmoidal) and software; there was evidence of an inadequate model for the flame-NAPE regression (*F* = 3.63; *P* = 0.0144).

#### Determination of enzyme selectivity

60 μl of Tris-HCl buffer was added to wells of a clear-bottom, black-walled, nontreated, 96-well plate, followed by 5 μl of Nape-pld (final concentration of 4.56 μg/ml), *A. oryzae* PLA1 (final concentration of 34.6 U/ml), or Tris-HCl buffer (negative control). About 5 μl of 7% (w/v) *N*-octyl-β-d-glucoside solution was added to the wells that received Nape-pld and 5 μl of Tris-HCl buffer to wells that received PLA1 or the negative control. After 1 h of incubation at 37°C, 10 μl of ice-cold substrate was added (final concentration of 4 μM), and fluorescence was measured as aforementioned. Background fluorescence in the absence of phospholipase was subtracted from all groups.

### Measurement of phospholipase activity in cells using flame-NAPE and PED-A1

HepG2, human embryonic kidney 293 (HEK293), 3T3-L1, and Caco-2 cells (human colon carcinoma cell lines) were purchased from ATCC (HB-8065, CRL-1573, CL-173, and HTB-37, respectively). Bone marrow progenitors were obtained from 8- to 12-week-old C57BL6/J mice. These progenitors were cultured using a previously published method to yield the bone marrow-derived macrophages (BMDMs) (32). The base cell media for all cell assays were DMEM with 4.5 g/l glucose, 1 mM sodium pyruvate, and 4 mM l-glutamine. For the growth of HEK293 and 3T3-L1 cells, phenol red and 10% (v/v) heat-inactivated FBS were added to base media. For Caco-2 cell growth, phenol red and 20% (v/v) heat-inactivated FBS were added to base media. For the assay, all cells were plated at 20,000 cells/well in clear-bottom, black-walled, tissue culture-treated, 96-well plates; negative control wells had no cells added. After the cells reached ∼90% confluency, the growth media were removed, and 100 μl/well of treatment media (see later) added, incubated for 1 h at 37°C, then 5 μl of 84 μM PED-A1 or flame NAPE in Tris-HCl/ethanol/DMSO (60:32:8 v/v/v) added to each of the wells (final concentration of 4 μM). For the primary HepG2 cell studies, the treatment media were base media with either NAPE-PLD inhibitor bithionol (Bith; final concentration of 15 μM), the pan-serine hydrolase lipase inhibitor tetrahydrolipstatin (THL; final concentration of 10 μM), both, or vehicle (DMSO); all contained 0.71% v/v DMSO final. For the supplemental HepG2 cell study, the treatment media were base media with either 15 μM Bith, 33 μM LEI-401 (Cayman Chemicals), both, or vehicle (DMSO); all contained 1.7% v/v DMSO final. For assays with HEK293, 3T3-L1, BMDM, or Caco-2 cells, the inhibitor media were base media with either Bith (final concentration of 15 μM) or vehicle (DMSO); with the concentration of DMSO in all assays being 0.016% v/v final. For all cellular experiments, fluorescence (excitation/emission = 488/530 nm) was measured at 1 min intervals in a BioTek Synergy H1 plate reader at 37°C. After the initial study in HepG2 cells, subsequent studies used the fluorescence values at 10 min for analysis. Final values were reported as substrate-normalized cellular fluorescence. To calculate this value, cellular fluorescence was first calculated as the fluorescence in the well measured at 10 min minus the average fluorescence of the wells with the appropriate substrate, but no cells (background fluorescence) were measured at 10 min. This cellular fluorescence value was then normalized by dividing this value by the average cellular fluorescence value for vehicle (DMSO only)-treated cells and multiplying by 100%. Each experiment was carried out on at least two separate days, and the normalized values from separate days were combined. For HepG2 cells, background fluorescence at 10 min for PED-A1 and flame-NAPE was typically 12% and 21%, respectively, of the raw fluorescence for vehicle-treated HepG2 cells. At the 10 min time point, the average raw fluorescence measured in vehicle-treated HepG2 cells with flame-NAPE was 66% that of similarly treated cells with PED-A1. For HEK293, 3T3-L1, BMDM, and Caco-2 cells where PED-A1 was used as substrate, the average background fluorescence (wells without cells) at the 10 min end point was 20%, 23%, 23%, and 19%, respectively, of the raw fluorescence of vehicle-treated cells. For HEK293, 3T3-L1, BMDM, and Caco-2 cells where flame-NAPE was used as substrate, the background fluorescence (wells without cells) at the 10 min end point was 24%, 34%, 33%, and 22%, respectively, of total raw fluorescence of vehicle-treated cells. The average raw fluorescence at 10 min end point for vehicle-treated HEK293, 3T3-L1, BMDM, and Caco-2 cells when 4 μM flame-NAPE was used as substrate was 89%, 94%, 76%, and 92%, respectively, of the raw fluorescence when 4 μM PED-A1 was used as substrate.

## Results

### Synthesis of flame-NAPE and characterization as NAPE-PLD substrate

**Fig. 2:**
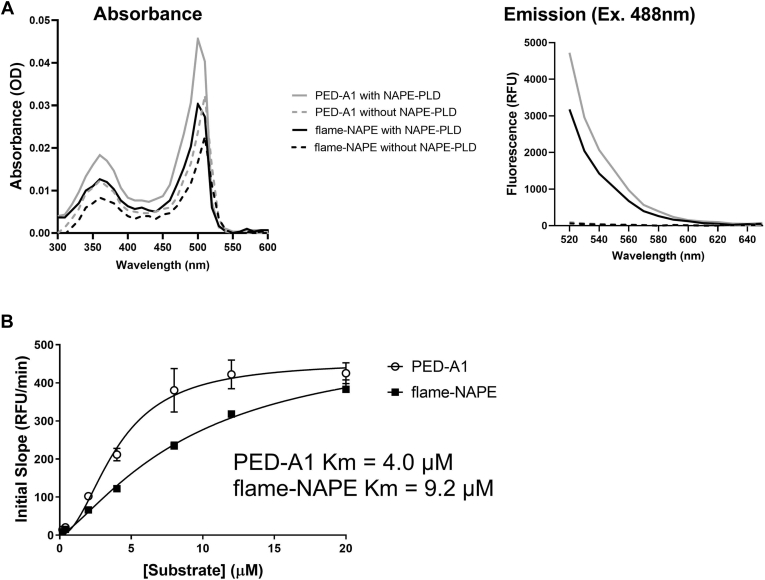
PED-A1 and flame-NAPE share similar spectrometric and Nape-pld utilization properties. A: UV-visible absorbance and fluorescence emission spectrum of PED-A1 and flame-NAPE with and without incubation with Nape-pld. Excitation at 488 nm was used to determine fluorescence emission spectrum. B: Michaelis-Menten plot for hydrolysis of PED-A1 (*K*_*M*_ = 4.0 μM) and flame-NAPE (*K*_*M*_ = 9.2 μM) by recombinant murine Nape-pld (4.56 μg/ml). Fluorescence excitation 488 nm and emission 530 nm. Each point represents mean ± SEM, n = 6.

To create a fluorogenic NAPE analog likely to be resistant to both PLA_1_ and PLA_2_ hydrolysis for our assay, we sought to generate a PED-A1 analog where the *sn*-1 ester bond of PED-A1 was substituted with either an ether bond or an *N*-methyl amide bond. Initial attempts to synthesize an ether bond analog gave poor results, so we focused our synthesis efforts on the *N*-methyl amide substitution. Details for synthesis of this *fl*uorogenic *am*ide NAPE analog (flame-NAPE; Fig. 1) are given in the supplemental data section. The absorbance spectrums of flame-NAPE closely matched those of PED-A1 both prior to and after hydrolysis with Nape-pld (Fig. 2A), confirming the presence of the BODIPY group (peak absorbance at 505 ± 3 nm) and the dinitrophenyl group (peak absorbance at 348 ± 4 nm) for flame-NAPE. Without hydrolysis with Nape-pld, no fluorescence emission was detected for either flame-NAPE or PED-A1 when exciting at 488 nm. Hydrolysis with Nape-pld resulted in similar fluorescence emission spectrum for flame-NAPE and PED-A1 (Fig. 2A). To assess the effect of the *N*-methyl amide bond substitution on substrate hydrolysis by Nape-pld, varying concentrations of flame-NAPE or PED-A1 were incubated with recombinant mouse Nape-pld, and the resulting rate of fluorescence generation was measured. These studies showed that the *K*_*m*_ of flame-NAPE (9.2 μM) was reasonably similar to that of PED-A1 (4.0 μM) (Fig. 2B), consistent with flame-NAPE being a robust Nape-pld substrate.

### Flame-NAPE is resistant to PLA_1_ hydrolysis in vitro

**Fig. 3:**
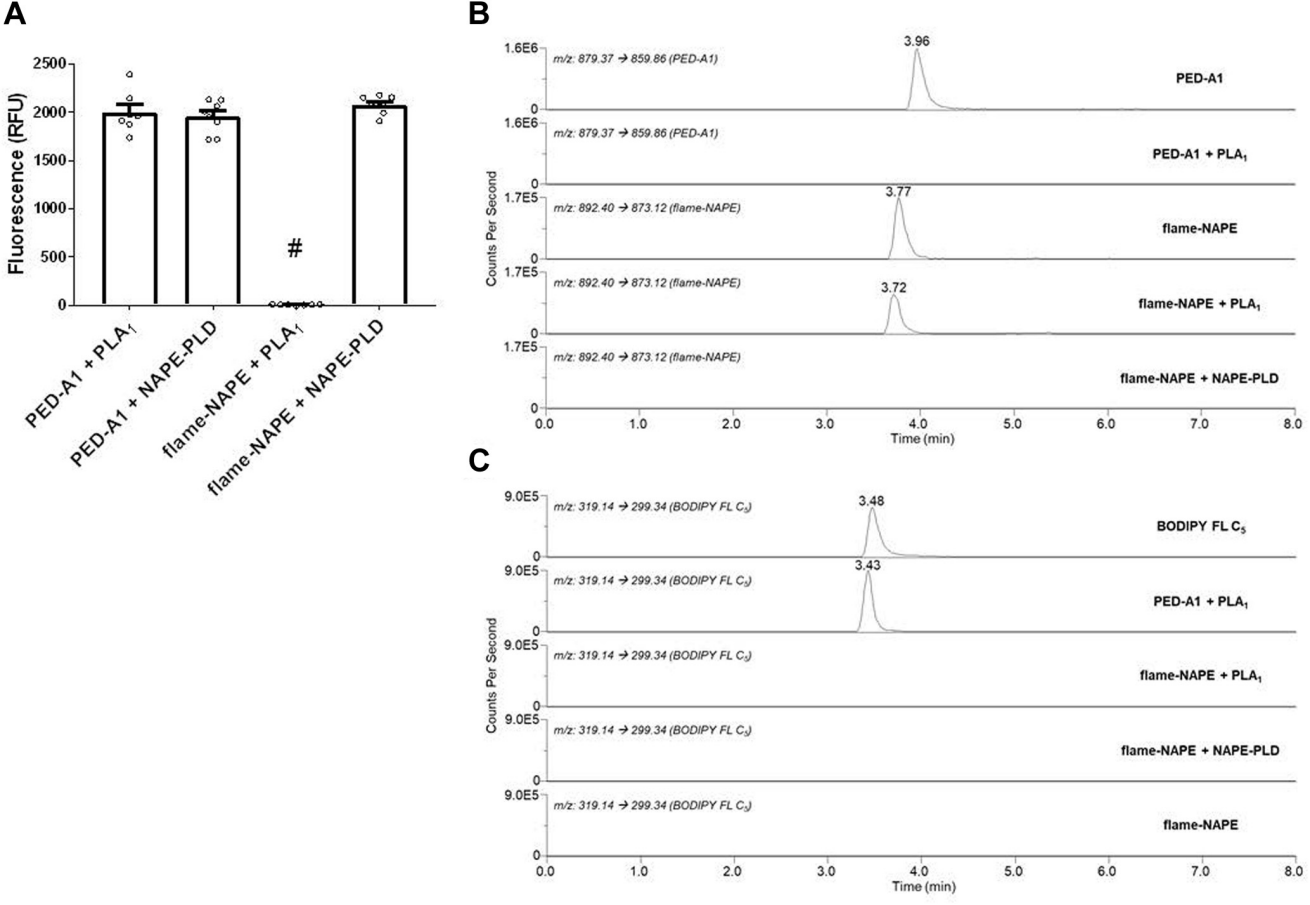
Flame-NAPE is a fluorogenic substrate for Nape-pld, but not Pla_1_, whereas PED-A1 is a fluorogenic substrate for both phospholipases. PED-A1 and flame-NAPE were incubated in vitro with either purified Pla_1_ from *Aspergillus oryzae* or recombinant murine Nape-pld, and the extent of hydrolysis was measured by fluorescence and LC/MS/MS. A: Fluorescence (excitation of 488 nm/emission of 530 nm) generated by phospholipase treatment with PED-A1 or flame-NAPE. One-way ANOVA; *P* = 0.0310; Tukey multiple comparisons, ^#^*P* < 0.0001 versus the other treatments. B: Representative multiple reaction monitoring chromatographs for unhydrolyzed PED-A1 (*m/z* 879.1→ 859.9) and unhydrolyzed flame-NAPE (*m/z* 892.4 → 873.1). C: Representative multiple reaction monitoring chromatographs for BODIPY FL C5 (*m/z* 319.1 → 299.3).

Resistance to hydrolysis by enzymes with PLA_1_-type activity would be a major advantage of flame-NAPE over the existing activity probe PED-A1. As expected, PED-A1 was readily hydrolyzed to a fluorescent product (excitation of 488/emission of 530 nm) by 50 min incubation with either purified *A. oryzae* Pla_1_ or recombinant Nape-pld (Fig. 3A). In contrast, flame-NAPE could be hydrolyzed to a fluorescent product only by Nape-pld and not by purified *A. oryzae* Pla_1_ (Fig. 3A). To confirm that the lack of fluorescence after incubation with Pla_1_ was the result of resistance of the substrate to hydrolysis, we developed an LC/MS/MS multiple reaction monitoring method to measure intact PED-A1 (*m/z* 879.4 → *m/z* 859.9) or flame-NAPE (*m/z* 892.4 → *m/z* 873.1) and the expected Pla_1_ hydrolysis product BODIPY-FL C_5_ (*m/z* 319.1 → *m/z* 299.3) and used this method to analyze the products of the aforementioned experiment. Incubation of PED-A1 with purified *A. oryzae* Pla_1_ for 50 min resulted in complete loss of LC/MS/MS signal for intact PED-A1 (Fig. 3B) and the formation of robust LC/MS/MS signal for BODIPY FL C_5_ (Fig. 3C). In contrast, incubation of flame-NAPE with purified *A. oryzae* Pla_1_ had little effect on the LC/MS/MS signal for intact flame-NAPE and failed to generate any signal for BODIPY FL C_5_ (Fig. 3C). Incubation of flame-NAPE with recombinant mouse NAPE-PLD resulted in complete loss of signal for intact flame-NAPE (and no formation of BODIPY FL C_5_), consistent with flame-NAPE being a selective Nape-pld substrate.

### Phospholipase activity assayed using flame-NAPE is insensitive to PLA_1_ inhibition

**Fig. 4:**
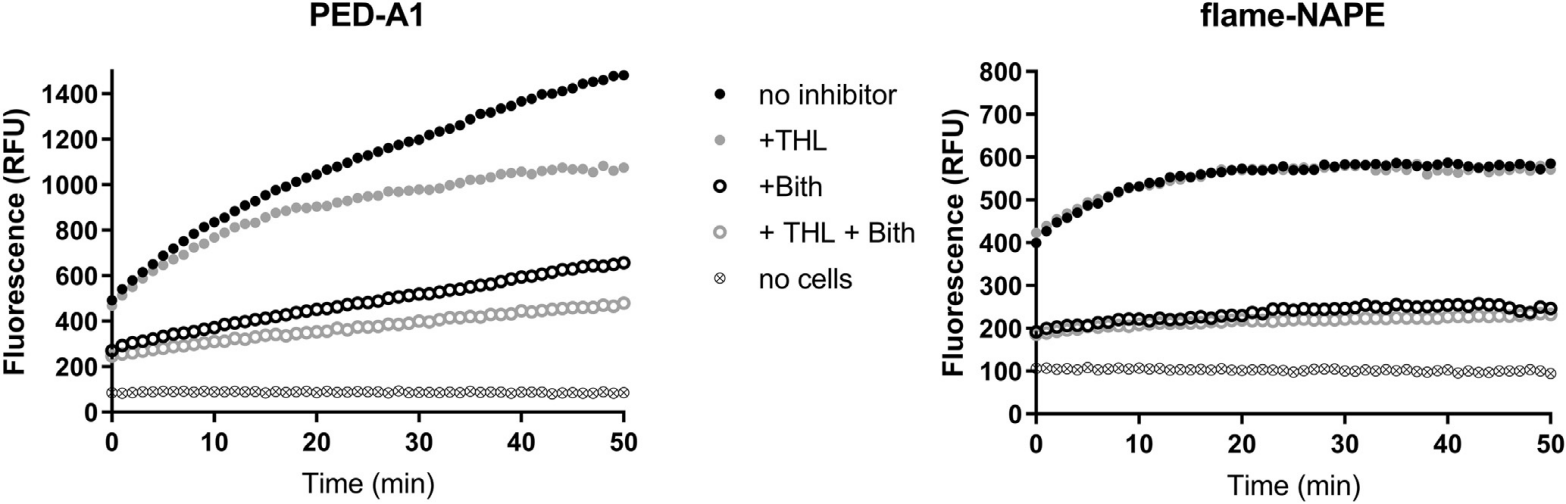
Phospholipase activity in HepG2 cells measured using flame-NAPE is sensitive to NAPE-PLD inhibition but not PLA_1_ inhibition, whereas phospholipase activity measured by PED-A1 is sensitive to both. HepG2 cells in 96-well plates were treated with 10 μM tetrahydrolipstatin (THL, a pan-lipase inhibitor) and/or 15 μM bithionol (Bith, an NAPE-PLD inhibitor) prior to the addition of either PED-A1 or flame-NAPE (4 μM). Representative 50 min fluorescence time course (1 read per minute) for each treatment. Similar time course obtained on two separate days. Symbols represent average of 6–8 replicate wells for each treatment.

In addition to their use to monitor NAPE-PLD activity in living cells, PED-A1 and PED6 have also been used extensively to measure PLA_1_ and PLA_2_ activity, respectively (22, 23, 24, 25, 26, 27, 28, 29, 30, 31). Therefore, fluorescence measured with these probes may reflect multiple phospholipase activities. HepG2 cells are a human hepatocellular carcinoma cell line that express both hepatic lipase (which has significant PLA_1_-type activity) and NAPE-PLD. To determine if using flame-NAPE in place of PED-A1 provided a more selective assay for NAPE-PLD activity in these cells, we investigated the sensitivity of the two substrates to inhibition of PLA_1_ and NAPE-PLD. THL covalently reacts with the catalytic serine of a broad range of lipases including PLA_1_s such as endothelial lipase and hepatic lipase (33, 34, 35, 36, 37). Because NAPE-PLD is a zinc metallohydrolase, rather than a serine hydrolase, THL does not inhibit the activity of this phospholipase. We recently identified Bith as a potent and selective NAPE-PLD inhibitor (27). In the absence of these inhibitors, addition of either 4 μM PED-A1 or 4 μM flame-NAPE to HepG2 cells generated robust time-dependent increases in cellular fluorescence (total fluorescence minus average fluorescence of substrate in media without cells) indicative of phospholipase activity (Fig. 4). The fluorescence measured at the 50 min time point using 4 μM PED-A1 was 2.9-fold greater than fluorescence measured using 4 μM flame-NAPE. Treatment with 10 μM THL decreased PED-A1 cellular fluorescence compared with no inhibitor, particularly at time points greater than 10 min (Fig. 4A). Treatment with 15 μM Bith also decreased PED-A1 cellular fluorescence, and treatment with THL in addition to Bith further decreased PED-A1 cellular fluorescence (Fig. 4A). In contrast, while treatment with 15 μM Bith markedly decreased flame-NAPE cellular fluorescence, treatment with 10 μM THL had no effect on flame-NAPE cellular fluorescence (Fig. 4B). These results are consistent with PED-A1 cellular fluorescence reporting both NAPE-PLD and PLA_1_ activity and flame-NAPE cellular fluorescence reporting NAPE-PLD activity but not PLA_1_ activity. These results also suggest that using a relatively short time point (e.g., 10 min) as an end point makes PED-A1 cellular fluorescence more selective for NAPE-PLD than longer time points (e.g., 50 min) where PLA_1_ activity much more significantly contributes to the signal.

The residual cellular fluorescence seen with flame-NAPE after 15 μM Bith treatment of the HepG2 cells (27% of vehicle-treated cells) likely reflects incomplete NAPE-PLD inhibition. The IC_95_ for Bith using recombinant Nape-pld is 62.0 μM (27), but 60 μM Bith is cytotoxic to HepG2 cells (supplemental Fig. S2). Residual flame-NAPE cellular fluorescence when combining 15 μM Bith with another NAPE-PLD inhibitor (33 μM LEI-401) (22) was only 10% (supplemental Fig. S3).

### Flame-NAPE can be used to selectively measure NAPE-PLD activity in multiple cell types

**Fig. 5:**
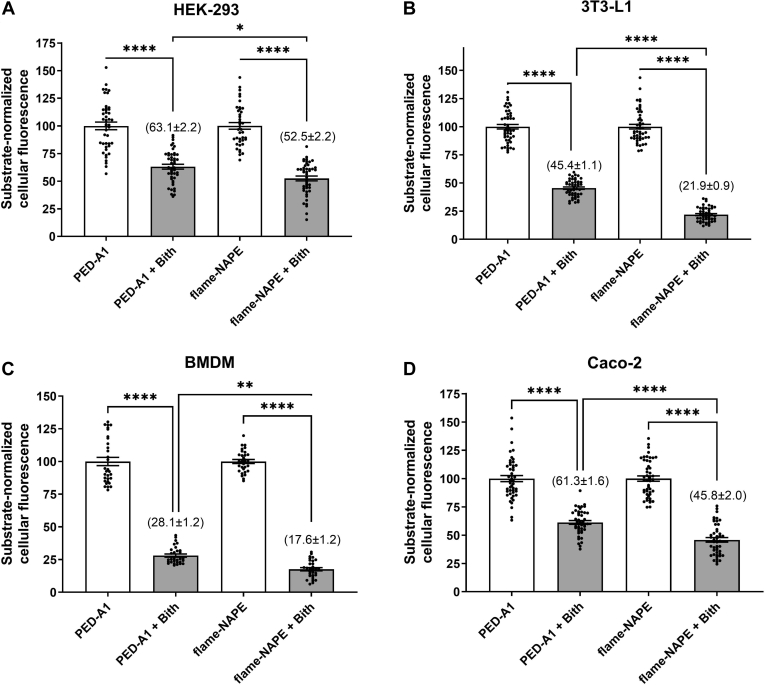
Phospholipase activity measured using flame-NAPE is more sensitive to NAPE-PLD inhibition than activity measured using PED-A1 in many cell types. Various cultured cell types were treated with or without 15 μM bithionol (Bith), an NAPE-PLD inhibitor, prior to incubation with PED-A1 or flame-NAPE (4 μM). Bars represent average ± SEM, n = 31–48 with 7–16 replicates each for three separate experimental days. A: HEK-293, (B) 3T3-L1, (C) BMDM (bone marrow-derived macrophage), and (D) Caco-2. One-way ANOVA for each cell type *P* < 0.0001, Tukey multiple comparisons test, ∗*P* < 0.025, ∗∗*P* < 0.0013, and ∗∗∗∗*P* < 0.0001. Number in parentheses equals substrate-normalized cellular fluorescence for Bith-treated cells (mean ± SEM).

NAPE-PLD has been implicated in playing key roles in adipocytes, kidney epithelial cells, intestinal enterocytes, and macrophages (7, 38, 39, 40, 41, 42). These cells express PLA_1_s in addition to NAPE-PLD (43, 44, 45, 46), so we sought to determine if flame-NAPE would report NAPE-PLD activity in these cell types more selectively than PED-A1. PED-A1 and flame-NAPE were incubated with cultured HEK293 epithelial cells, 3T3-L1 cells (mouse preadipocyte cell line), mouse BMDMs, and Caco-2 that had been pretreated with or without 15 μM Bith, and cellular fluorescence was analyzed at the 10 min time point (Fig. 5). In all four cell types, Bith inhibited flame-NAPE cellular fluorescence to a greater extent than PED-A1 cellular fluorescence (47% vs. 37% inhibition in HEK293%; 78% vs. 55% in 3T3-L1; 82% vs. 72% in BMDM; and 54% vs. 39% in Caco-2). These results are consistent with flame-NAPE cellular fluorescence selectively reporting NAPE-PLD activity and PED-A1 cellular fluorescence reporting the combined activity of NAPE-PLD and PLA_1_.

## Discussion

Our results demonstrate that the use of flame-NAPE provides a facile method to selectively assay NAPE-PLD activity in a variety of cell types. Such an assay has long been needed to enable studies elucidating how various external stimuli and signaling pathways modulate NAPE-PLD activity and NAE biosynthesis. Understanding control of NAE biosynthesis is critical because NAEs including AEA, PEA, and OEA regulate a host of physiological processes, including feeding behavior, nociception, leukocyte infiltration, insulin secretion, and macrophage polarization (4, 5, 6, 7, 8, 9, 10, 11); therefore, reduced activity of their biosynthetic enzymes may be important factors in disease.

Understanding how NAE biosynthesis is regulated and why it becomes dysregulated under some conditions has been challenging in part because of the lack of appropriate probes and associated methods to measure the activity of individual NAE biosynthetic enzymes. In addition to NAPE-PLD, at least two other enzymes act on NAPE. ABHD4 acts as a PLA_1_ and PLA_2_ to convert NAPE to GP-NAE (19), which can be converted to NAE by GDE1 or GDE4 or other similar phosphodiesterases (6, 15, 16). Genetic deletion of *Abhd4* in mice demonstrates that this NAE biosynthetic enzyme plays a critical role in developmental anoikis (47). *Abhd4* deletion in mice does not significantly decrease their brain levels of AEA, OEA, PEA, or their precursor NAPEs, but does reduce levels of GP-NAEs (48). To the best of our knowledge, the effects of *Abhd4* deletion on NAE levels in tissues other than brain have not been reported. Another alternative pathway for NAE biosynthesis utilizes an unidentified NAPE-hydrolyzing phospholipase C (NAPE-PLC) that hydrolyzes NAPE to diacylglycerol and phospho-NAE. Phospho-NAE can then be converted to NAE via phosphatases such as PTPN22 (49). Lipopolysaccharide stimulation of macrophages decreases expression of NAPE-PLD (20, 49) and reduces levels of PEA (20, 50) and OEA (50) but increases levels of AEA (49, 50, 51) and phospho-AEA (49). These results suggest that unlike NAPE-PLD, the putative NAPE-PLC preferentially utilizes *N*-arachidonyl-PE over *N*-palmitoyl-PE or *N*-oleoyl-PE and might therefore control AEA levels. However, genetic deletion of *Nape-pld* does reduce levels of AEA as well as PEA and OEA in brain (52, 53), so control of AEA biosynthesis likely depends on multiple pathways (54). Therefore, the straightforward assay method to measure NAPE-PLD activity we report here should be helpful in better understanding how this enzyme controls levels of AEA, OEA, and PEA.

Fluorogenic activity assays have considerable advantages in terms of reduced time and resource consumption compared with methods such as TLC and LC/MS/MS, as long as the probes utilized selectively measure the desired activity. While the fluorogenic probes PED6 and PED-A1 have been used in high-throughput screening to identify NAPE-PLD inhibitors in vitro (22, 27, 28), they are sensitive to hydrolysis by other phospholipases likely to be present in cells and tissues. PED6 was originally created as a probe for PLA_2_ activity (23), whereas PED-A1 was created as a probe for PLA_1_ activity (26). Since ABHD4 has both PLA_1_ and PLA_2_ activity, neither PED-A1 nor PED6 can readily distinguish between NAPE-PLD and ABHD4 activity. Many other PLA_1_s or PLA_2_s that can hydrolyze PED-A1 and PED6 are present in cells and tissue where measurement of NAPE-PLD activity is of interest. For instance, PED-A1 has been used to measure the PLA_1_ activity of endothelial lipase in human umbilical vein endothelial cells (26). Other mammalian PLA_1_s include hepatic lipase, phosphatidylserine-specific PLA_1_, membrane-associated phosphatidic acid-selective PLA_1_α/β, pancreatic lipase-related protein 2, and phospholipase A/acyltransferase-2 (55, 56). Our studies with THL, a pan-inhibitor of serine hydrolase-type lipases, confirm that a significant portion of PED-A1 fluorescence measured in cultured cells results from phospholipase activity other than NAPE-PLD activity, particularly at longer end points. THL markedly reduced PED-A1 fluorescence in HepG2 cells, while having no effect on flame-NAPE fluorescence. Furthermore, in all the cell types we tested, the NAPE-PLD inhibitor (Bith) decreased PED-A1 cellular fluorescence to a lesser extent than it decreased flame-NAPE cellular fluorescence. Thus, if PED-A1 (or PED6) fluorescence is used to measure NAPE-PLD activity in cultured cells or tissues, THL (or another appropriate inhibitor) should be added prior to initiation of the assay to block serine hydrolase-type lipases, and the end point of the assay should be relatively short (i.e., 10 min).

Use of flame-NAPE to measure NAPE-PLD activity in our assay method overcomes the major limitations of PED-A1 and PED6. Substituting an *N*-methyl amide group at the *sn*-1 ester bond made flame-NAPE completely resistant to hydrolysis by *A. oryzae* Pla_1_, unlike PED-A1 that was entirely hydrolyzed by this enzyme. Flame-NAPE fluorescence was not inhibited by THL in HepG2 cells, supporting the notion that serine hydrolase-type phospholipases do not significantly contribute to flame-NAPE hydrolysis. Flame-NAPE fluorescence was significantly inhibited by 15 μM Bith in all the cell types we tested. It is possible that in some cell types, a nonserine hydrolase-type phospholipase such as the putative NAPE-PLC contributes to flame-NAPE cellular fluorescence. One limitation of our validation studies is that the contribution of the putative NAPE-PLC to flame-NAPE fluorescence cannot be tested directly because it has not been cloned or purified. However, significant NAPE-PLC contribution seems unlikely because this putative NAPE-PLC appears to prefer *N*-arachidonyl-PE over NAPEs with shorter saturated *N*-acyl chains (49), likely making flame-NAPE a poor NAPE-PLC substrate.

Although the method we report here is to measure relative NAPE-PLD activity in cultured cells, the previous use of PED6 in zebrafish embryo and larvae to image sites of active pla_2_ in the digestive tract (25, 57) suggest that flame-NAPE could be used in a similar manner for imaging active nape-pld. This might be particularly useful for examining the effects of diet and other environmental factors on intestinal, adipocyte, or macrophage nape-pld activity. Imaging of the flame-NAPE could also provide insight into changes in NAPE-PLD localization within cultured cells, although potential diffusion of the BODIPY-phosphatidic acid from its initial site of formation must be considered.

In summary, using flame-NAPE provides a rapid and straightforward method to selectively assay NAPE-PLD activity that should facilitate studies to identify factors that alter NAPE-PLD activity, the role of NAPE-PLD activity in controlling individual levels of NAEs, whether changes in NAPE-PLD activity contribute to disease, and the extent to which potential interventions rescue NAPE-PLD activity.

## Data availability

Processed data generated during this study are included in the published article and its supplemental data files. The raw and processed data for these studies are archived at the FigShare repository Web site https://doi.org/10.6084/m9.figshare.17035610.

## Supplemental data

This article contains supplemental data (58, 59, 60).

## Conflict of interest

The authors declare that they have no conflicts of interest with the contents of this article.
