# Supplementary material for: Selective measurement of NAPE-PLD activity via a PLA1/2-resistant fluorogenic N-acyl-phosphatidylethanolamine analog
Source: J Lipid Res. 2021 Nov 26;63(1):100156. doi: 10.1016/j.jlr.2021.100156 (PMC8953660; doi:10.1016/j.jlr.2021.100156)
Supplement: Supplemental data [file mmc1.docx]

**Supplemental Information**

**1. General Procedure**: All non-aqueous reactions were performed in flame-dried or oven dried round-bottomed flasks under an atmosphere of argon. Stainless steel syringes or cannula were used to transfer air- and moisture-sensitive liquids. Reaction temperatures were controlled using a thermocouple thermometer and analog hotplate stirrer and monitored using liquid-in-glass thermometers. Reactions were conducted at room temperature (approximately 21-23 °C) unless otherwise noted. Flash column chromatography was conducted using silica gel 230-400 mesh. Reactions were monitored by analytical thin-layer chromatography, using EMD Silica Gel 60 F254 glass-backed pre-coated silica gel plates. The plates were visualized with UV light (254 nm) and stained with potassium permanganate or *p*- anisaldehyde-sulfuric acid followed by charring. Yields were reported as isolated, spectroscopically pure (1) compounds.

**2. Materials**: Solvents and chemicals were purchased from Sigma-Aldrich, Acros Organics, TCI and/or Alfa Aesar and used without further purification. Solvents were purchased from Fisher Scientific. Dry dichloromethane (CH_2_Cl_2_) was collected from an MBraun MB-SPS solvent system. Dichloroethane (DCE) was distilled from calcium hydride and storedover 4 Å molecular sieves. Triethylamine, N,N-dimethylformamide (DMF) and dimethyl sulfoxide (DMSO) were used as received in a bottle with a Sure/Seal. N,N-diisopropylethylamine was distilled from calcium hydride and stored over KOH. BF_3_·Et_2_O was distilled prior to use from calcium hydride. Deuterated solvents were purchased from Cambridge Isotope Laboratories.

**3. Instrumentation**: Preparative reverse phase HPLC (Gilson) was performed using a Phenomenex Gemini column (5 micron, 110 Å, 50 x 21.20 mm, flow rate 30 mL/min) with UV/Vis detection. Infrared spectra were obtained as thin films on NaCl plates using a Thermo Electron IR100 series instrument and are reported in terms of frequency of absorbance (cm^-1^). ^1^H NMR spectra were recorded on Bruker 400 or 600 MHz spectrometers and are reported relative to internal chloroform (^1^H, δ 7.26), methanol (^1^H, δ 3.31), and DMSO (^1^H, δ 2.50). Data for ^1^H NMR spectra are reported as follows: chemical shift (δ ppm), multiplicity (s = singlet, d = doublet, t = triplet, dd = doublet of doublet, ddd = doublet of doublet of doublet, m = multiplet, br=broad), coupling constants (Hz), and integration. ^13^C NMR were recorded on Bruker 100 or 150 MHz spectrometers and are reported relative to internal chloroform (^13^C, δ 77.1), methanol (^13^C, δ 49.2), and DMSO (^13^C, δ 40.3). Low-resolution mass spectra were acquired on an Agilent Technologies Series 1200 single quad ChemStation autosampler system using electrospray ionization (ESI) in positive mode. High-resolution mass spectra (HRMS) were obtained from the Department of Chemistry and Biochemistry, University of Notre Dame Mass Spectrometry Center or the Mass Spectrometry Research Center at Vanderbilt University.

**4. Chemical Synthesis of flame-NAPE**

**
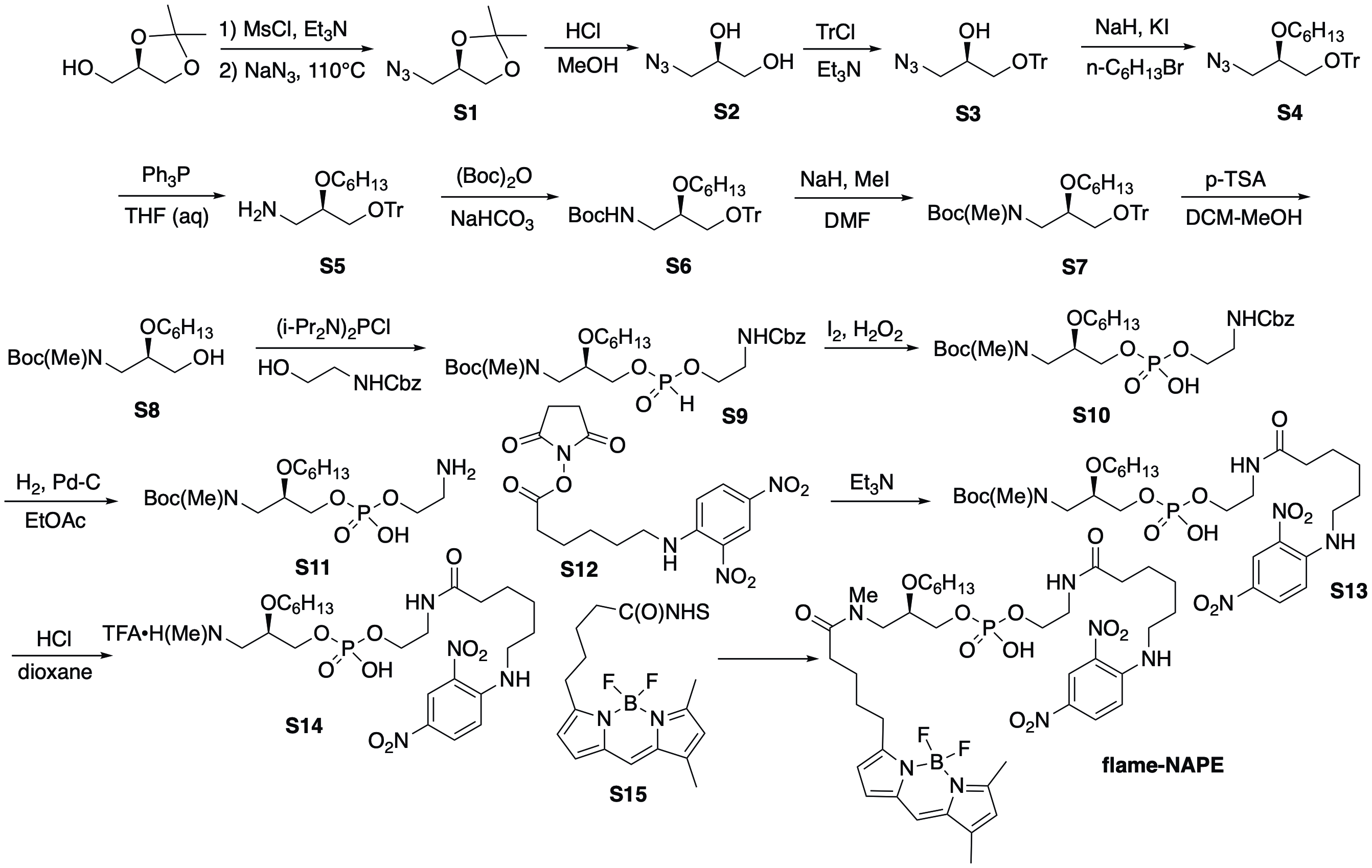
**

**Supplemental Figure 1**

**(R)-3-azidopropane-1,2-diol (S2)** To a solution of (R)-4-(azidomethyl)-2,2-dimethyl-1,3-dioxolane (**S1**)^1^ (2.03 g, 12.9 mmol) in anhydrous MeOH (60 mL) at room temperature was added 4M HCl in 1,4-dioxane (484 µL, 1.93 mmol). The resulting mixture was stirred at room temperature for 2 h, and then concentrated. The residue was dissolved in dichloromethane, dried over Na_2_SO_4_, and filtered. The filtrate was concentrated and dried under vacuum to provide diol **S2**, which was used in the next step without further purification. Compound characterization data was consistent with previous report.^2^

**(R)-1-azido-3-(trityloxy)propan-2-ol (S3)** To a solution of (R)-3-azidopropane-1,2-diol (**S2**) (1.51 g, 12.9 mmol) in DMF (12 mL) at room temperature were added Et_3_N (5.39 mL, 38.7 mmol), DMAP (78.8 mg, 0.65 mmol), and (chloromethanetriyl)tribenzene (5.39 g, 19.3 mmol) sequentially. The resulting mixture was stirred at room temperature overnight, then MeOH (10 mL) was added and stirring was continued for another 20 min. The solvent was removed under reduced pressure. To the residue was added diethyl ether (80 mL), and the mixture was filtered and concentrated. The residue was purified by column chromatography (0-60% ethyl acetate in hexane) to provide ether **S3** (4.0 g, 86% over two steps). Compound characterization data was consistent with previous report.^2^

**(R)-((3-azido-2-(hexyloxy)propoxy)methanetriyl)tribenzene (S4)** To a solution of (R)-1-azido-3-(trityloxy)propan-2-ol (**S3**) (3.7 g, 10.3 mmol) in DMF (20 mL) at 0 ˚C was added 60% NaH in mineral oil (0.66 g, 16.5 mmol). The resulting mixture was stirred for 15 min, then 1-bromohexane (1.9 mL, 13.4 mmol), and KI (342.0 mg, 2.06 mmol) were added. The reaction mixture was allowed to warm to room temperature and stirred overnight. Then the reaction was quenched with saturated aqueous NH_4_Cl (50 mL) and the mixture was extracted with ethyl acetate (3 X 70 mL). The combined organic layers were dried over Na_2_SO_4_, filtered, and concentrated. The residue was purified by column chromatography (0-60% ethyl acetate in hexane) to provide ether **S4** (3.1 g, 68%). MS(ES) *m/z* = 443.59/466.5 [M+Na] ^+^; LCMS RT = 1.88 min.

**tert-butyl (R)-(2-(hexyloxy)-3-(trityloxy)propyl)carbamate (S6)** To a solution of (R)-((3-azido-2-(hexyloxy)propoxy)methanetriyl)tribenzene (**S4**) (960.0 mg, 2.16 mmol) in THF (21 mL) were added water (2.1 mL) and PPh_3_ (851.5 mg, 3.25 mmol). The resulting mixture was stirred in a sealed tube at 55 °C for 16 h, then was allowed to cool to room temperature. Di-tert-butyl dicarbonate (566.8 mg, 2.60 mmol), NaHCO_3_ (363.6 mg, 4.32 mmol), and water (1 mL) were added. The resulting mixture was stirred at room temperature for 4 h, and then extracted with ethyl acetate (3 X 30 mL). The combined organic layers were dried over Na_2_SO_4_, filtered, and concentrated. The residue was purified by column chromatography (0-30% ethyl acetate in hexane) to provide carbamate **S6** (1.05 g, 93%). ^1^H NMR (400 MHz, CDCl_3_) δ 7.46 (m, 6H), 7.30 (m, 6H), 7.23 (m, 3H), 4.80 (brs, 1H), 3.47 (m, 4H), 3.17 (m, 3H), 1.43 (s, 9H), 1.29 (m, 8H), 0.89 (t, *J* = 6.8 Hz, 3H). MS(ES) *m/z* = 517.71/540.6 [M+Na] ^+^; LCMS RT = 1.85 min.

**tert-butyl (R)-(2-(hexyloxy)-3-(trityloxy)propyl)(methyl)carbamate (S7)** To a solution of tert-butyl (R)-(2-(hexyloxy)-3-(trityloxy)propyl)carbamate (**S6**) (1.05 g, 2.03 mmol) in DMF (8 mL) at 0 °C was added 60% NaH in mineral oil (105.0 mg, 2.64 mmol). After stirring at 0 °C for 15 min, MeI was added. The resulting mixture was allowed to warm to room temperature slowly and stirred for 16 h. The reaction was quenched with saturated aqueous NH_4_Cl (30 mL), and the mixture was extracted with ethyl acetate (3 X 30 mL). The combined organic layers were dried over Na_2_SO_4_, filtered, and concentrated. The crude was used in the next step without further purification**.** MS(ES) *m/z* = 531.74/554.6 [M+Na] ^+^; LCMS RT = 1.92 min.

**tert-butyl (R)-(2-(hexyloxy)-3-hydroxypropyl)(methyl)carbamate (S8)** To a solution of tert-butyl (R)-(2-(hexyloxy)-3-(trityloxy)propyl)(methyl)carbamate (**S7**) (1.08 g, 2.03 mmol) in a mixture of dichloromethane (8 mL) and MeOH (12 mL) at room temperature was added p-toluenesulfonic acid monohydrate (38.6 mg, 0.2 mmol). The resulting mixture was stirred for 3 h, and then was diluted with ethyl acetate (80 mL) and washed with saturated aqueous NaHCO_3_ (20 mL). The organic layer was dried over Na_2_SO_4_, filtered, and concentrated. The residue was purified by column chromatography (0-50% ethyl acetate in hexane) to provide alcohol **S8** (550.0 mg, 93%).^1^H NMR (400 MHz, CDCl_3_) δ 3.68 (brs, 1H), 3.51 (m, 6H), 3.28 (dd, *J* = 4.8, 14.4 Hz, 1H), 2.93 (s, 3H), 1.56 (m, 2H), 1.47 (s, 9H), 1.32 (m, 6H), 0.89 (t, *J* = 6.8 Hz, 3H). MS(ES) *m/z* = 289.42/312.4 [M+Na] ^+^; LCMS RT = 1.25 min.

**tert-butyl ((2R)-3-(((2-(((benzyloxy)carbonyl)amino)ethoxy)(hydroxy)phosphaneyl)oxy)-2-(hexyloxy)propyl)(methyl)carbamate (S9)** To a suspension of 1-chloro-N,N,N',N'-tetraisopropylphosphanediamine (250.0 mg, 0.94 mmol) in diethyl ether (6.5 mL) at 0 °C was added benzyl(2-hydroxyethyl)carbamate (**S8**) (186.8 mg, 0.96 mmol) and Et_3_N (134 µL, 0.96 mmol) as a solution in dichloromethane (6.5 mL). The mixture was stirred at 0 °C for 2 h, and then concentrated. To the residue was added diethyl ether (10 mL), and the resulting mixture was filtered. The filtrate was concentrated and dried under vacuum. The residue was dissolved in a mixture of dichloromethane (3.5 mL) and acetonitrile (3.5 mL). To this solution was added tert-butyl (R)-(2-(hexyloxy)-3-hydroxypropyl)(methyl)carbamate (90.0 mg, 0.31 mmol). The mixture was stirred at room temperature for 10 min, and then 1H-tetrazole (35.0 mg, 0.5 mmol) was added. Stirring was continued for 2 h, and then another batch of tert-butyl (R)-(2-(hexyloxy)-3-hydroxypropyl)(methyl)carbamate (90.0 mg, 0.31 mmol) was added and followed by addition of 1H-tetrazole (30.0 mg, 0.43 mmol). The resulting mixture was allowed to stir at room temperature overnight, and then concentrated. The residue was dissolved in ethyl acetate (50 mL) and washed with saturated aqueous NaHCO_3_ (15 mL). The organic layer was dried over Na_2_SO_4_, filtered, and concentrated. The residue was purified by column chromatography (0-30% ethyl acetate in hexane) to provide a mixture (170 mg) of the desired product and inseparable impurities. This mixture was further purified by preparative HPLC (50-95% acetonitrile in water, 0.1% TFA) to phosphite **S9** (118.0 mg, 35%). (400 MHz, CDCl_3_) δ 7.79 (s, 0.5H), 7.35 (m, 5H), 6.92 (d, *J* = 716 Hz, 0.5H), 6.88 (d, *J* = 716 Hz, 0.5H), 5.12 (s, 2H), 4.18 (m, 3H), 4.00 (m, 1H), 3.50 (m, 7H), 2.91 (s, 3H), 1.55 (m, 2H), 1.46 (s, 9H), 1.28 (m, 6H), 0.89 (t, *J* = 6.8 Hz, 3H). MS(ES) *m/z* = 530.6/553.6 [M+Na] ^+^; LCMS RT = 1.37 min.

**tert-butyl ((2R)-3-(((2-(((benzyloxy)carbonyl)amino)ethoxy)(hydroxy)phosphoryl)oxy)-2-(hexyloxy)propyl)(methyl)carbamate (S10)** To a solution of phosphite **S9** (118.0 mg, 0.22 mmol) in dichloromethane (2.5 mL) at room temperature was added iodine (5.6 mg, 0.22 mmol) and 30% H_2_O_2_ (68 µL, 0.67 mmol). The mixture was stirred overnight, and then diluted with ethyl acetate (30 mL). The organic layer was washed with 20% aqueous Na_2_S_2_O_3_, dried over Na_2_SO_4_, filtered, and concentrated. The residue was purified by column chromatography (0-30% MeOH in dichloromethane) to provide phosphonate **S10** (90.0 mg, 74%). (400 MHz, CDCl_3_) δ 7.28 (m, 5H), 5.04 (s, 2H), 3.81 (m, 4H), 3.51 (m, 3H), 3.26 (m, 6H), 2.84 (s, 3H), 1.43 (m, 2H), 1.41 (s, 9H), 1.23 (m, 6H), 0.86 (t, *J* = 6.8 Hz, 3H). MS(ES) *m/z* = 546.6/569.6 [M+Na] ^+^; LCMS RT = 1.24 min.

**tert-butyl ((2R)-3-(((2-(((benzyloxy)carbonyl)amino)ethoxy)(hydroxy)phosphoryl)oxy)-2-(hexyloxy)propyl)(methyl)carbamate (S11)** To a solution of carbamate **S10** (45.0 mg, 82.3 µmol) in ethyl acetate (10 mL) was added 10% palladium on carbon (22.0 mg, 20.6 µmol). The resulting mixture was shaken in a sealed bottle under 50 psi hydrogen gas atmospheres at room temperature on a Parr shaker for 16 h. The mixture was filtered through a celite plug, which was washed with ethyl acetate (3 X 5 mL). The combined filtrate and washings were concentrated and dried under vacuum to provide amine **S11** (25.0 mg, 74%), which was used in the next step without further purification. MS(ES) *m/z* = 412.46/413.6 [M+H] ^+^; LCMS RT = 0.94 min.

**tert-butyl ((2R)-3-(((2-(6-((2,4-dinitrophenyl)amino)hexanamido)ethoxy)(hydroxy)-phosphoryl)oxy)-2-(hexyloxy)propyl)(methyl)carbamate (S13)** To a solution of tert-butyl ((2R)-3-(((2-aminoethoxy)(hydroxy)phosphoryl)oxy)-2-(hexyloxy)propyl)(methyl)carbamate (**S11**) (25.0mg, 60.6 µmol) in dichloromethane (2 mL) at 0 ˚C was added Et_3_N (25 µL, 0.18 mmol) and 2,5-dioxopyrrolidin-1-yl 6-((2,4-dinitrophenyl)amino)hexanoate (40.0 mg, 0.1 mmol). The ice bath was removed and the reaction mixture was allowed to warm to room temperature and stirred overnight. Ethyl acetate (15 mL) was added and the mixture was washed with 0.5 M HCl (3 mL), water (3 mmL), and brine (5 mL). The organic layer was dried over Na_2_SO_4_, filtered, and concentrated. The residue was purified by column chromatography (0-30% MeOH in dichloromethane) to provide amide **S13** (38.5 mg, 91%). (400 MHz, CDCl_3_) δ 9.11 (d, *J* = 2.4 Hz, 1H), 8.57 (brs, 1H), 8.27 (dd, *J* = 1.6, 9.2 Hz, 1H), 6.95 (d, *J* = 9.6 Hz, 1H), 3.92 (m, 3H), 3.63 (m, 2H), 3.43 (m, 7H), 2.91 (s, 3H), 2.25 (m, 2H), 1.74 (m, 4H), 1.49 (m, 2H), 1.44 (s, 9H), 1.28 (m, 8H), 0.87 (t, *J* = 6.8 Hz, 3H). MS(ES) *m/z* = 691.72/692.72 [M+H] ^+^; LCMS RT = 1.27 min.

**2-(6-((2,4-dinitrophenyl)amino)hexanamido)ethyl ((R)-2-(hexyloxy)-3-(methylamino)propyl) hydrogen phosphate (S14)** To a solution of carbamate **S13** (38.5 mg, 55.7 µmol) in dichloromethane (3 mL) at 0 ˚C was added 4 M HCl in 1,4-dioxane (209 µL, 0.83 mmol). The mixture was stirred for 4 h, and then concentrated and dried under vacuum to provide amine **S14** (37.0 mg, quantitative) as a hydrochloride salt. The crude product was purified by 8 mg by preparative HPLC (5-95% acetonitrile in water, 0.1% TFA) to provide 3.6 mg of **S14** as TFA salt, which was used in the next step. (400 MHz, CDCl_3_) δ 9.03 (d, *J* = 2.4 Hz, 1H), 8.29 (dd, *J* = 2.4, 9.6 Hz, 1H), 7.17 (d, *J* = 9.6 Hz, 1H), 4.17 (m, 1H), 4.06 (m, 3H), 3.85 (m, 1H), 3.71 (m, 1H), 3.50 (m, 5H), 3.21 (m, 2H), 2.75 (s, 3H), 2.26 (t, *J* = 6.8 Hz, 2H), 1.77 (m, 2H), 1.70 (m, 2H), 1.63 (m, 2H), 1.50 (m, 2H), 1.33 (m, 6H), 0.90 (t, *J* = 6.8 Hz, 3H). MS(ES) *m/z* = 591.6/592.6 [M+H] ^+^; LCMS RT = 1.04 min.

**Flame-NAPE** To a solution of TFA salt **S14** (3.6 mg, 5.1 µmol) in DMF (0.5 mL) was added 0.5 M TEAB (0.25 mL). Then 2,5-dioxopyrrolidin-1-yl 5-(5,5-difluoro-7,9-dimethyl-5H-4l4,5l4-dipyrrolo[1,2-c:2',1'-f][1,3,2]diazaborinin-3-yl)pentanoate (3.0 mg, 7.1 µmol) was added in one portion. The mixture was sonicated to make the solid fully dissolve, and then stirred at room temperature in dark for 2 h. The solvent removed under reduced pressure (water bath < 40 ˚C) and the residue was purified by column chromatography (10-50% MeOH/CHCl_3_,1% water) to provide **flame-NAPE** (2.0 mg, 44%). (400 MHz, CDCl_3_) δ 9.03 (s, 1H), 8.50 (brs, 1H), 8.15 (d, *J* = 8.8 Hz, 1H), 7.01 (s, 1H), 6.86 (m, 2H), 6.25 (d, *J* = 4.0 Hz, 1H), 6.06 (s, 1H), 3.95 (m, 3H), 3.71 (m, 2H), 3.51 (m, 3H), 3.34 (m, 3H), 3.09 (s, 3H), 2.92 (m, 2H), 2.49 (s, 3H), 2.38 (m, 2H), 2.26 (m, 2H), 2.21 (s, 3H), 1.74 (m, 10H), 1.45 (m, 4H), 1.24 (m, 6H), 0.86 (t, *J* = 6.8 Hz, 3H). MS(ES) *m/z* = 893.73/916.7 [M+Na] ^+^; LCMS RT = 1.37 min. ^3^

**Supplemental Figure 2. Cytotoxicity of bithionol (Bith) for HepG2 cells.** HepG2 cells were cultured for 24 h in presence of various concentrations of Bith and cell viability measured using 3-(4,5-dimethylthiazol-2-yl)-2,5-diphenyltetrazolium bromide) (MTT). All values were normalized to vehicle only controls. 1-way ANOVA p<0.0001, Tukey’s multiple comparisons test, ****p<0.0001 compared to 0 μM concentration.

**Supplemental Figure 3. Inhibition of flame-NAPE cellular fluorescence by NAPE-PLD inhibitors.** HepG2 cells were cultured with vehicle (DMSO), 33 uM LEI-401, 15 uM Bith, or both LEI-401 and Bith and cellular fluorescence with flame-NAPE measured. 1-way ANOVA p<0.0001, Tukey’s multiple comparisons test, ****p<0.0001. Mean ± SEM.

**References**

1. Kalhor-Monfared S, Beauvineau C, Scherman D, Girard C. Synthesis and cytotoxicity evaluation of aryl triazolic derivatives and their hydroxymethine homologues against B16 melanoma cell line. European Journal of Medicinal Chemistry. 2016;122:436-41. doi: 10.1016/j.ejmech.2016.06.057. PubMed PMID: WOS:000383003900037.

2. Doboszewski B, Groaz E, Herdewijn P. Synthesis of Phosphonoglycine Backbone Units for the Development of Phosphono Peptide Nucleic Acids. European Journal of Organic Chemistry. 2013;2013(22):4804-15. doi: 10.1002/ejoc.201300523. PubMed PMID: WOS:000331293500014.

3. Ferguson, C, Prestwich G, Madan D. Fluorogenic assay for lysophospholipase D acivity using fluorogenic lysophospholipid derivatives as substrates, and diagnostic and screening applications. United States, US20100260682 A1
